# Wavelength and orientation dependent capture of light by diatom frustule nanostructures

**DOI:** 10.1038/srep17403

**Published:** 2015-12-02

**Authors:** Julien Romann, Jean-Christophe Valmalette, Matilde Skogen Chauton, Gabriella Tranell, Mari-Ann Einarsrud, Olav Vadstein

**Affiliations:** 1 Department of Material Science and Engineering, Norwegian University of Science and Technology NTNU, NO-7491 Trondheim, Norway; 2 IM2NP UMR 7334 CNRS, Université de Toulon, P.O. Box 20132, 83957 La Garde Cedex, France; 3 Department of Biotechnology, Norwegian University of Science and Technology NTNU, NO-7491 Trondheim, Norway

## Abstract

The ecological success of diatoms is emphasized by regular blooms of many different species in all aquatic systems, but the reason behind their success is not fully understood. A special feature of the diatom cell is the frustule, a nano-patterned cell encasement made of amorphous biosilica. The optical properties of a cleaned single valve (one half of a frustule) from the diatom *Coscinodiscus centralis* were studied using confocal micro-spectroscopy. A photonic crystal function in the frustule was observed, and analysis of the hyperspectral mapping revealed an enhancement of transmitted light around 636 and 663 nm. These wavelengths match the absorption maxima of chlorophyll a and c, respectively. Additionally, we demonstrate that a highly efficient light trapping mechanism occurred, resulting from strong asymmetry between the cribrum and foramen pseudo-periodic structures. This effect may prevent transmitted light from being backscattered and in turn enhance the light absorption. Based on our results, we hypothesize that the multi-scaled layered structure of the frustule improves photosynthetic efficiency by these three mechanisms. The optical properties of the frustule described here may contribute to the ecological success of diatoms in both lentic and marine ecosystems, and should be studies further *in vivo*.

Diatom frustules are one of the most complex biosilica nanostructures found in nature, and in living cells the frustules may function as protective cases with high mechanical stability^1^,^2^,^3^. Gas exchange and virus control are also potential roles of the frustules, and the porous microstructures were shown to affect the movement of particles on the surface, possibly introducing selectivity in terms of nutrients or particle transport into the cells^4^. The ecological success of diatoms is related to their ability to rapidly acclimate and adapt their light-harvesting apparatus to varying light conditions in the aquatic environment^5^, and efficient carbon dioxide concentration mechanism^6^. The biological function of frustules is not yet fully understood, but several authors have suggested that the frustules improve light harvesting in diatoms, thereby contributing to the photosynthetic efficiency^7^,^8^.

Hence, optical properties of diatom frustules is a field of increasing interest considering the biosilica structure functions as a photonic crystal with waveguide properties^9^,^10^,^11^. Photonic crystals, consisting of periodic changes in refractive index (silica/surrounding medium), modulate the passing light, and are the cause of color in butterfly wings or antireflection in insect eyes, to mention some examples from nature. Photonic crystal properties have been proposed for frustules of *Coscinodiscus granii*^12^ and a light focusing effect was detected in *C. wailesii* frustule valves^13^. A dependence between the spatial distribution of light transmitted through a single *C. wailesii* frustule and the wavelength has recently been reported^14^. The study reported interesting simulation results, suggesting light confinement effects under the diffraction limit of a lens with the dimensions of a frustule. We have recently described light convergence and trapping effects in valves from *C. wailesii* and *C. centralis*^15^. These outstanding properties have led to many ideas of using frustules in optical applications including micro-optics elements, optical sensors, photonic devices, solar cells enhancers and plasmonic devices, biosensing, replication templating, drug delivery and microfluidics^16^,^17^,^18^,^19^,^20^,^21^.

Here we report bi-dimensional (2D) hyperspectral mapping measurements on a single valve using a confocal system, showing how *C. centralis* valves trap light and modify its spectrum to match the optical absorption of the specific chlorophyll *a* and *c* photoreceptors in living diatoms. Our data point to the outstanding match between the optical properties of the valves and two aspects of the ecological success of diatoms: light harvesting and photosynthesis. This work also provides a direct visualization of the light trapping effect induced by the frustule structure within the diatom.

## Results

### Valve structure

**Figure 1:**
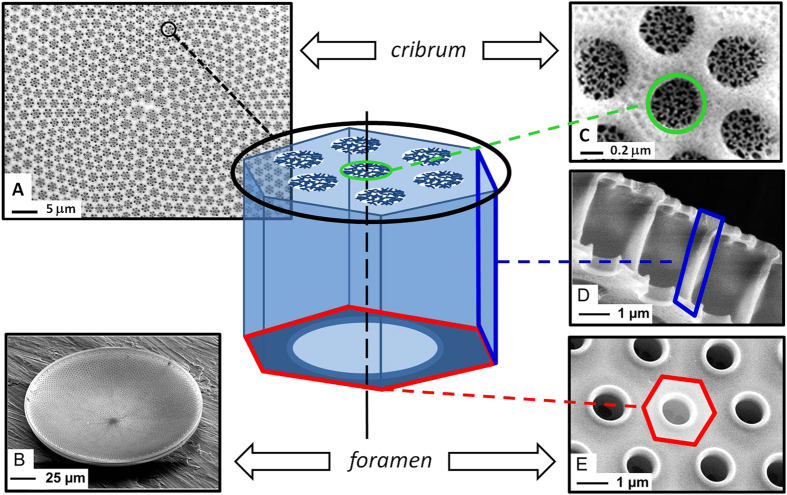
General scheme of *Coscinodiscus centralis* bio-silica valve and corresponding SEM images of cribrum side (**A**), foramen side pointing upwards (**B**), details of cribrum (**C**), areola side walls (**D**) and foramen (**E**).

Cleaned frustule valves featured several overlapping nanoporous patterns (Fig. 1A). A single valve can be considered as the superposition of three layers (Fig. 1B). The layer on the external side of the valve (cribrum) showed a multi-scale structure of flower-like micro porous patterns with hexagonal symmetry (Fig. 1C). The cribrum layer is about 200 nm thick, and each pore of the flower-like pattern is subdivided into several smaller pores by thin silica branches (cribellum). The areola can be described as a honeycomb structure (about 200 nm thick and 2.5 μm high) perpendicular to the cribrum (Fig. 1D). The flower-like, porous pattern of the cribrum is centered above one cavity defined by the honeycomb structure of the areola. The third layer is called the foramen and defines the internal side of the valve (Fig. 1E), and displays a hexagonal array of circular pores with a diameter of about 1 μm. The porous structure of the foramen matches the structure of the other layers, making each pore of the foramen centered with both a hexagonal cavity of the areola and a flower-like porous pattern of the cribrum.

### Spectral modification of transmitted light

**Figure 2:**
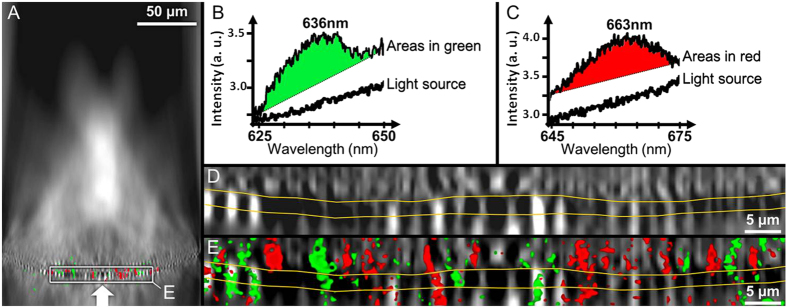
Hyperspectral analysis of a single valve of *Coscinodiscus centralis* oriented with the foramen side upwards and the direction of the incident light indicated by a white arrow (**A**). The wavelength regions (green and red) where transmission spectra (**B,C**) differing from the light source are detected. Close view of the hyper-map in A (**D**). Close view of the composite image in A (**E**). The yellow lines in (**D,E**) indicate the transition between the foramen and cribrum layers of the valve, with the honeycombed structure in the middle.

The hyper-map showed a non-uniform distribution of transmitted light along the mapping plane (Fig. 2A) and a substantial convergence and consequently concentration of light towards the internal side of the frustule. The observed intensity distribution and the radial symmetry of the valve suggest multiple light cones transmitted through the valve, similar to a multifocal lens. The spectrum of the light source was linear within the wavelength range of observation. However, two distinct spectral bands were identified in the transmitted light. These spectral bands are centered at 636 nm (Fig. 2B) and 663 nm (Fig. 2C) and match the absorption maxima of chlorophyll *c* (631–636 nm) and chlorophyll *a* (662–675 nm), respectively^(26–29)^. Spectral analysis of the hyper-map in Fig. 2A showed that these bands were only found in micron and sub-micron scaled zones, all localized within a narrow region centered on the valve (indicated by an arrow in Fig. 2A). A magnified hyper-map of this narrow region (Fig. 2A, insert) revealed alternating pattern of high and low intensities, around and within the valve structure (Fig. 2D). The spectral differentiation mostly occurred within the high intensity regions, and more frequently above the foramen than within or below the valve structure (Fig. 2E).

### Orientation dependent optical measurements

Diatoms like *C. centralis* are freely suspended in sea water and submitted to a random incident light orientation. Because the valve structure (Fig. 1) consists of two different sides (cribrum and foramen) we therefore investigated illumination angle and side dependent properties.

**Figure 3:**
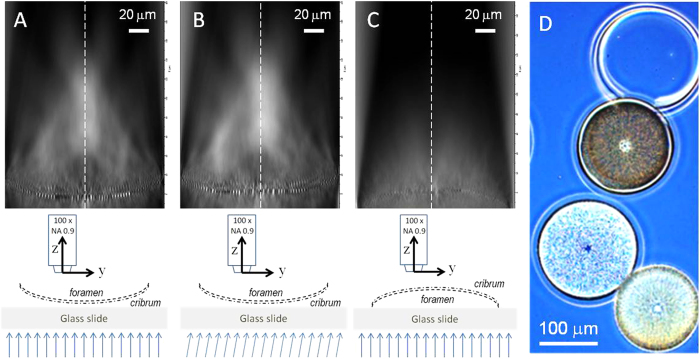
Hyper-maps of a single valve of *Coscinodiscus centralis* under different orientations. The cribrum faces a normal incident light (**A**). The cribrum faces a 10° tilted incident light (**B**). The foramen faces a normal incident light (**C**). Light microscopy picture of cleaned valves, where the effect of orientation is visible as light valve (like in **A**, cribrum side facing the incoming light (like in **A,B**) and a dark valve (like in (**C**), foramen facing the incoming light) (**D**). In each map, the spectral envelope is normalized to the light source intensity. The schemes describe the measurement configurations. The vertical dotted lines indicate the symmetry axis of the valve (S). The scale bar (20 μm) is the same for the Figure (**A–C**).

Figure 3 shows the intensity distribution of the transmitted light, integrated over the spectral range 400–800 nm with normal (Fig. 3A,C) and 10° left-tilted (Fig. 3B) incident parallel illumination. The objective axis was oriented normal to the surface of the frustule, and in both measurements the transmitted light showed a higher intensity along the symmetry axis (S). There was also a “focus-like” effect (normal to the valve) independent of the incident angle. However, more detailed observation of the light distribution revealed three significant modifications induced by the 10° left-tilt angle: First, the intensity on the right side of the symmetry axis S was significantly higher than on the left side, leading to the loss of the axial symmetry. Second, the intensity increased drastically in the left area above the frustule, while right area remained unchanged. Third, the total intensity was higher than under normal incident illumination.

To investigate the light propagation from the interior to the exterior of the frustule the frustule was placed in the reversed orientation, i.e. illumination of the foramen side (Fig. 3). The intensity scale was fixed to the same value in all images, to compare light propagating in the two directions over the valve. Furthermore, each pixel is directly proportional to the intensity of light collected under 128° by the 100 x objective. As a result, black pixels means that either no light pass this the region or the light propagates under a higher angle. No focusing effect was observed when light was going through the frustule from the foramen to the cribrum side (Fig. 3C) and the total intensity above the frustule (or “light leakage”) was very weak compared to the light transmitted into the frustule (Fig. 3A,B). The trapping effect was visible in normal light microscopy when observing frustules oriented in the two different ways (Fig. 3D).

## Discussion

The ecological success of diatoms is explained by the photophysiology and adaptive properties of the living cell, but the potential role of the biosilica frustules is seldom discussed in relation to light propagation and absorption by the pigments. The different layers with pores of different diameters and patterns in the frustule of *C. centralis* (Fig. 1) appeared to be an effective light trapping and modulating structure (i.e. a photonic waveguide). These properties may play a direct role in improving the conditions for efficient photosynthesis in the living cells. For the organism, there is a metabolic cost associated with frustule construction, involving uptake of silica, vacuoles for transport and construction, and synthesis of highly specialized proteins that are involved in the process^22^. However, the data presented here showed that the construction and patterning of the frustule may to some extent compensate for the costs associated with the silica cell encasement, by selectively enhancing propagation of optimal wavelengths and focusing the light towards the chloroplasts.

The observed spectral changes (Fig. 2) match the maximum absorption specific to chlorophyll *a* and *c*, the two main photosynthetic pigments found in diatoms. Light absorption and photosynthesis occur in the chloroplasts, and in centric diatoms such as *Coscinodiscus* the numerous, small chloroplasts are distributed towards the cell surface. From an optical point of view, this localization close to the frustule means that the chloroplasts are optically coupled to the light propagating through the biosilica structure (18). Yamanaka *et al.* (2008) suggested that the photonic structures functioned to reduce blue light absorption, because excessive blue light may lead to formation of reactive oxygen.

Optical properties of such complex structures as frustules have been studied using computational methods^11^,^12^. The symmetry of the diatom frustule structure can be roughly described as 1D pseudo-periodic hexagonal pattern in the radial direction, but mismatch along this direction leads to a higher order of symmetry (Fig. 1A). This 2D sunflower-like symmetry is also called phyllotactic spirals, and the optical properties of such structures has been simulated by summation of a large number of small periodic areas arranged with a radial symmetry^15^.

Based on theoretical modelling it has been suggested that frustules increase light backscattering and attenuation compared to naked cells^23^. The intensity mappings presented in this study showed a selective transmission of light from the outside to the inside of the valve (Fig. 3). Our study of the relation between incident light angle and valve orientation show some interesting features with respect to the intensity distribution of the transmitted light. The hyper-maps showed a symmetrical distribution of the transmitted light around the symmetry axis of the valve when the incoming light was normal to the valve. With the incident light tilted by an angle of about 10° we observed some clear effects. The direction of transmitted light was unchanged, indicating that once the photons travel within the frustule they are guided by the material, independent of the angle of the incident light. We also observed a substantial light concentration effect along the symmetry axis, and the light intensity was higher on the same side as the incoming light. This light concentration results from a convergence phenomenon, in agreement with observations reported in a previous work presenting one-dimensional intensity measurements^13^. These three effects cannot be explained by a classic diffraction phenomenon and strongly support the presence of optical waveguide properties emerging from the nanoporous structure of the valve^15^.

From our measurements we see that the light was directed towards the center somewhere above the valve. Measured from the pictures in Fig. 3, the focal region is approximately 50–55 μm long and with a focusing spot about 50 μm above the valve inside. The height of the whole frustule is approximately 70 μm (Fig. 1), so the effect occurs within the height of a whole *C. centralis* cell and towards the opposite valve. The central part of large centric diatoms is commonly occupied by a liquid-filled vacuole, an organelle which functions in e.g. ion exchange and to maintain osmotic cell pressure. Moreover, our data indicate that light propagating to the inside of the valve is held back within the frustule (Fig. 3C). If the alternating patterns of holes (with low refractive index) and silica structure (with high refractive index) work as a waveguide, incoming light should be focused somewhere above the valve whereas light travelling the opposite way will be scattered. This differential effect of light modulation and transmittance from the outside to the inside (vs. from the inside to the outside) of the valve can explain the observations seen in Fig. 3D, where different orientation of the valves lead to differences in light transmission through the valve. Measurements of fluorescence from the valves verified that they were clean from organic compounds (Romann *et al.*, submitted to Phycologia), and the apparent difference in color is probably related to the light confinement effect that happens on the interior side of the valve. For the living diatom, this effect helps to minimize the loss of light that is on the inside, and increases the probability of light absorption within the chloroplasts.

Albeit our data are from a single, cleaned valve in air, it is tempting to hypothesize that these effects of spectral modification, directional light concentration and light blocking effects, also occur in intact, living cells. However, *in vivo* experiments of living algae suspended in sea water are difficult to conduct. Indeed, the investigation of light propagation inside the frustules requires their disassembling by chemical routes leading to cell alteration and removal of organic matter from the surface. Similar observations have been made in other species of *Coscinodiscus*^13^,^14^,^15^.

The results presented here are of importance for the use of nanostructured biosilica frustules in applications, but the results also contribute to an increased biological understanding of living diatoms and what contributes to their fitness and thus ecological success. Any potential drawbacks of synthesizing and wearing such a complex cell encasement may be compensated by the photonic modulator properties of the frustule that enhances light absorption and ultimately photosynthesis. We hypothesize that the nanostructure of the frustule improves photosynthetic efficiency by focusing the light and optimizing spectral quality when passing through the frustule, and by the confinement of the transmitted light in the frustule. This hypothesis represents a new mechanism to explain the ecological and evolutionary success of diatoms. Studies on intact diatoms in water would be a logical start for further studies to shed more light – literally – on the ecological implications of diatom frustules.

## Methods

Diatoms identified as *Coscinodiscus centralis* were collected by net haul in the Trondheim fjord (Norway, 63°29’N, 10°15’E) and cleaned as follows: Diatoms were filtered out of the culture medium, washed 3 times in Milli-Q water, centrifuged at 4500 rpm for 10 min between each washing and dried overnight at 60 °C. Dried diatom valves (2 mg) were placed in hydrogen peroxide (H_2_O_2_, 10 mL) solution (30%) and stirred at 90 °C for 24 h. After addition of HCl solution (37%, 1 mL) the sample was centrifuged at 4500 rpm for 10 min. Finally, the cleaned frustules were rinsed with Milli-Q water, centrifuged (4500 rpm, 10 min) 3 times and stored in ethanol (96%). Valves and girdle bands became disassembled during the cleaning process due to the removal of organic material and the mechanical effects of centrifugation (Romann *et al.*, submitted to Phycologia). Cleaned valves were deposited on quartz substrate, and investigated using a Zeiss Ultra 55 FE Scanning Electron Microscopy (SEM) operated at 15 kV.

A single frustule valve mounted with the inside (foramen) upwards on quartz substrate was analyzed using a custom-made optical setup, involving a confocal micro-spectrometer. A tungsten halogen light source illuminated the sample from below, using an optical fiber with a diameter of 1000 μm. A motorized stage allowed bidirectional motion of the sample in the plane of the quartz substrate. The transmitted light was collected by a 100 × objective (NA 0.9) mounted on a vertically moving motorized stage. The objective was aligned with the central axis of the optical fiber, and the collection volume was therefore located in an area where the incident beam was assumed to be parallel coming from below the frustule. The collected light was directed through a confocal aperture (50 μm), and dispersed on a CCD detector by a grating. Hyperspectral maps showing the spatial distribution of transmission spectra were acquired through a single frustule valve, with each spectrum being a single pixel (1 × 1 μm) of a hyper-map. The greyscale used on the hyper-maps corresponds to the integrated intensities of the acquired transmission spectra over a wavelength range. All spectra were acquired within a wavelength range from 400 to 800 nm. All maps were acquired along the [YZ] plane, which is normal to the substrate and intersects the center of the valve.

This technique is powerful to investigate the light distribution but we must keep in mind that, due to the collection angle of the objective, only a fraction of the total light can be detected, i.e. the fraction of light with angle lower than 64°. Moreover, the light analyzed in a confocal volume localized below the frustule plane is strongly scattered after crossing the frustule layer, hence this below-part of the hyper-map image will not be discussed in this paper.

## Additional Information

**How to cite this article**: Romann, J. *et al.* Wavelength and orientation dependent capture of light by diatom frustule nanostructures. *Sci. Rep.* **5**, 17403; doi: 10.1038/srep17403 (2015).
